# Lipidemic Profile of Patients with Non-Small Cell Lung Cancer and Its Association with Driver Mutations: A Tertiary Center Retrospective Study

**DOI:** 10.3390/cancers18030374

**Published:** 2026-01-25

**Authors:** Maria Lagadinou, Dimitrios Efthymiou, Fotios Sampsonas, Prokopis Karidis, Ioanna Marlafeka, Eirini Adamopoulou, Christos Michailides, Pinelopi Bosgana, Ourania Papaioannou, Emmanouil Psarros, Panagiota Tsiri, Vasilina Sotiropoulou, Matthaios Katsaras, Vasiliki Tzelepi, Argyrios Tzouvelekis, Markos Marangos

**Affiliations:** 1 Department of Internal Medicine, Medical School, University Hospital of Patras, 265 04 Patras, Greecedr.jomarl@gmail.com (I.M.);; 2 Division of Infectious Diseases, University Hospital of Patras, 265 04 Patras, Greece; 3 Respiratory Medicine Department, Medical School, University of Patras, 265 04 Patras, Greece; ouraniapapaioannou@outlook.com (O.P.);; 4 Department of Pathology, Agios Andreas Hospital of Patras, 265 04 Patras, Greece; 5 Department of Pathology, University Hospital of Patras, 265 04 Patras, Greece; btzelepi@upatras.gr

**Keywords:** triglycerides, lung cancer, total cholesterol, adenocarcinoma, squamous cell carcinoma

## Abstract

Background: Altered lipid metabolism has been reported in several malignancies, but its clinical relevance in non-small cell lung cancer (NSCLC) remains uncertain. This study aimed to compare serum lipid parameters between NSCLC patients and healthy controls and to explore their association with histological subtype and selected driver mutations. Methods: We retrospectively analyzed serum total cholesterol (TC), low-density lipoprotein (LDL), high-density lipoprotein (HDL), and triglycerides (TG) in patients diagnosed with adenocarcinoma or squamous cell carcinoma from 2021 to 2024, alongside a control group of 100 healthy individuals. Statistical comparisons were performed using appropriate parametric or nonparametric tests after normality assessment (Shapiro–Wilk), and *p*-values were adjusted using the Benjamini–Hochberg false discovery rate (FDR). Results: A total of 160 NSCLC patients were included. Most were male (75.5%) and current or former smokers (96.1%), with a mean age of 70.4 ± 10.3 years. Squamous cell carcinoma was the predominant subtype (64.4%). Hypocholesterolemia was observed in 59.9% of patients, while hypercholesterolemia was less frequent (40.1%). Compared with controls, patients had significantly lower HDL levels (*p* = 0.007, FDR-adjusted *p* = 0.024), while other lipid markers showed no statistically significant differences after correction for multiple testing. Differences between adenocarcinoma and squamous cell carcinoma were not statistically significant. Squamous cell carcinoma patients had higher TG but lower TC, LDL, and HDL levels compared with adenocarcinoma. A negative correlation between TG and ROS1 expression remained significant (r = −0.223, FDR-adjusted *p* = 0.004). Conclusions: In this retrospective, real-world cohort, only HDL levels demonstrated a robust difference between NSCLC patients and controls. Observed associations should be interpreted cautiously due to potential confounding factors and incomplete clinical data inherent to retrospective analyses. Prospective studies are needed to clarify whether lipid alterations play a biological or prognostic role in NSCLC.

## Simple Summary

This study investigates whether routine blood lipid measurements, such as cholesterol and triglycerides, differ in people with non-small cell lung cancer compared with healthy individuals, and whether these measurements relate to tumor type or key genetic changes that guide modern treatments. Because these tests are inexpensive and widely available, identifying consistent patterns could support future research on simple markers that reflect tumor biology. We reviewed medical records from a tertiary hospital and compared blood lipid levels in lung cancer patients with those of matched controls, and we also examined differences between the main lung cancer subtypes and selected tumor gene alterations. We found that high-density lipoprotein was consistently lower in lung cancer patients, was lower in squamous tumors than in adenocarcinomas, and was lower in patients who died during follow-up. Triglyceride levels showed an inverse association with a specific gene alteration. These findings highlight lipid changes as potential research targets and support larger prospective studies.

## 1. Introduction

Lipid disorders are among the most common metabolic abnormalities linked to cardiovascular disease. Dysregulation of lipid metabolism has emerged as a hallmark of malignant transformation, affecting multiple processes including membrane biogenesis, energy production, oxidative stress response, and oncogenic signaling [1,2,3]. Altered lipid uptake, storage, and regulation of proteins and enzymes involved in lipid metabolism have also been documented in several cancers. In lung cancer, one of the most prominent changes is the reprogramming of cellular metabolism [4].

Lung cancer remains one of the most prevalent malignancies worldwide, accounting for approximately 13% of all cancer diagnoses [5,6], and continues to be the leading cause of cancer-related death in both men and women [7]. Early diagnosis and treatment can improve survival outcomes; however, most NSCLC patients are diagnosed at advanced stages, when curative options are limited. The identification of druggable oncogenic driver mutations has markedly improved clinical outcomes in recent years, leading to the development of targeted therapies and personalized treatment approaches [8]. Despite these advances, prognosis remains poor, underscoring the need for new biomarkers that can facilitate earlier diagnosis, refine risk stratification, and guide therapeutic decision-making.

To sustain uncontrolled growth and proliferation, cancer cells remodel the metabolic pathways of carbohydrates, amino acids, and lipids. These adaptations enable them to meet the energy and biosynthetic demands of rapid cell division while evading signals that would otherwise induce growth arrest or apoptosis [4]. Among these metabolic shifts, alterations in lipid metabolism have recently attracted increasing attention [8]. Lipids are not only structural components of cellular membranes but also act as signaling molecules and energy reservoirs, influencing tumor progression, immune evasion, and metastatic potential.

One of the most prominent features of lung cancer cells is the reprogramming of cellular metabolism, enabling them to sustain uncontrolled growth and adapt to the tumor microenvironment. Cancer cells undergo profound remodeling of carbohydrate, amino acid, and lipid metabolic pathways [9,10,11]. Among these metabolic shifts, lipid metabolism has recently attracted increasing attention, both as a potential biomarker source and as a therapeutic target [2].

Given these insights, investigating serum lipid profiles in lung cancer may provide valuable information on tumor biology and disease progression. Lipid parameters are inexpensive and routinely measured in clinical practice, making them attractive candidates for biomarkers of tumor metabolic state or prognosis [12]. Furthermore, histological subtypes of lung cancer, particularly adenocarcinoma (ADC) and squamous cell carcinoma (SCC), may exhibit distinct metabolic signatures, including differences in lipid metabolism, that could inform personalized approaches to diagnosis and therapy [13,14].

The objective of our study was to evaluate serum levels of total cholesterol (TC), low-density lipoprotein (LDL), high-density lipoprotein (HDL), and triglycerides (TG) in patients with squamous cell carcinoma and adenocarcinoma of the lung. We further aimed to assess the frequency of dyslipidemia in these patients, compare their lipid profiles with those of healthy controls, and explore potential correlations between lipid levels and biomarker expression. Additionally, we aimed to examine whether specific histological subtypes and oncogenic drivers are associated with distinct lipid alterations, which may reflect underlying differences in tumor metabolism and biology.

## 2. Patients and Methods

### 2.1. Data Collection

This was a single-center, retrospective, observational study using real-world data obtained anonymously from the medical records of the Respiratory Department and the pathological archives of the Pathology Department at Patras University Hospital. Data collection covered the period from 1 January 2021, when molecular testing for driver mutations was first implemented, through 31 December 2024, as already described [15].

All patients were identified through the hospital’s tumor registry and laboratory information systems, and relevant clinical data were cross-referenced to ensure completeness and accuracy. Extracted variables included demographic characteristics (age at diagnosis, sex), clinical data (smoking status, histological subtype [adenocarcinoma, squamous cell carcinoma], and date of diagnosis), laboratory data, as well as molecular and biomarker information. Data cleaning and verification were performed by two independent investigators to minimize transcription errors and ensure data integrity. Cases with incomplete lipid or molecular testing were excluded from comparative analyses but retained for descriptive statistics when appropriate.

Driver mutation analysis included testing for EGFR (del19, L858R, T790M), ALK fusions, and ROS1, using a real-time PCR platform. DNA was isolated from FFPE tissue with the Roche Cobas DNA Sample Preparation Kit (Roche Hellas, Athens, Greece). Mutation testing was performed on the Cobas z480 analyzer with real-time PCR assays supplied by Roche Diagnostics. PD-L1 expression, when available, was assessed by immunohistochemistry using the 22C3 antibody on the Dako Link48 Autostainer (Agilent Dako, Sweden) [16,17]. All molecular tests were requested at the discretion of the treating physicians (oncologists and pulmonologists) in accordance with ESMO guidelines [18]. These tests were typically prescribed for patients with advanced or locally advanced NSCLC, as well as for those with early-stage disease who were not suitable candidates for surgery. In certain cases of sqNSCLC, molecular testing was also performed in younger patients or in individuals without a recent or significant smoking history.

The lipid panel included total cholesterol (TC), high-density lipoprotein (HDL), low-density lipoprotein (LDL), and triglycerides (TG), measured using standardized enzymatic methods in the hospital’s central laboratory. Serum TC < 160 mg/dL was defined as hypocholesterolemia and TC > 200 mg/dL as hypercholesterolemia. TG > 150 mg/dL indicated hypertriglyceridemia and LDL > 100 mg/dL was classified as borderline high. Fasting status at the time of sampling was not systematically recorded, reflecting real-world clinical practice, but samples were typically obtained during morning hours. Although recent epidemiological studies have demonstrated an association between thyroid hormone levels and cancer risk, data on thyroid function were not available in the present study [19].

### 2.2. Study Population

All adult patients who were referred to Patras University Hospital, the biggest hospital in western Greece, with newly diagnosed squamous cell carcinoma or adenocarcinoma of the lung and available medical records were included. Data collected included age, sex, smoking status, and serum lipid levels measured at or within six months prior to diagnosis. Patients were excluded from analysis if they had incomplete molecular data, missing lipid panel measurements, a previous history of other active concurrent malignancies or were receiving a lipid-lowering medication. A control group of 100 healthy individuals without cancer, matched for sex and age, was included in comparison with the study group. These individuals were not hospital inpatients and were not evaluated for acute illness. Controls were matched to the lung cancer group by sex and age distribution (±5 years), and the same exclusion criteria were also applied. Exact individual-level matching was not feasible due to the retrospective design, but demographic frequencies were aligned during sampling. No data for performance status, BMI and socioeconomic background were available and therefore were not collected.

All data were anonymized and entered into an Excel database. This study was approved by the Scientific, Research, and Ethics Committee of Patras University Hospital (Ref. No. 399/5 September 2024).

### 2.3. Statistical Analyses

Statistical analysis was conducted using SPSS version 25 (IBM SPSS Statistics 25). Data were presented as mean ± standard deviation (SD) for continuous variables and as absolute numbers and percentages for categorical variables. A *p*-value of less than 0.05 was considered statistically significant. Pearson’s correlation coefficient was employed to examine relationships between continuous variables, while Spearman’s rank correlation was additionally used for non-normally distributed data. All serum lipid parameters (TC, LDL, HDL, TG) were examined for normality using the Shapiro–Wilk test, separately for each comparison group. Normally distributed variables (*p* > 0.05 in all groups) were compared using the *t*-test, while non-normally distributed variables were compared using the Mann–Whitney U test. Categorical variables were compared using the χ^2^ test. To account for the four lipid parameters across two primary comparisons (cancer vs. controls; adenocarcinoma vs. squamous), Benjamini–Hochberg FDR correction was applied.

Subgroup analyses were performed according to histological subtype and molecular biomarker status to explore potential interactions between lipid parameters and tumor characteristics. For each lipid parameter, the study performed two primary comparisons: 1. Lung cancer vs. control group and 2. Adenocarcinoma vs. squamous cell carcinoma. To evaluate whether lipid levels differed according to survival status (alive vs. deceased), *t*-test was used following verification of equal variances with Levene’s test.

## 3. Results

### 3.1. Lipid Levels and Lung Cancer

Between January 2021 and December 2024, 462 patients with adenocarcinoma and squamous cell lung cancer were identified, of whom 160 had complete data and were included in the final analysis. Most of them were male (76.9%, *n* = 123) and current or former smokers (95.6%, *n* = 153), with a mean age of 70.4 ± 10.3 years. Squamous cell carcinoma was the most common cancer subtype (64.4%). Most patients were diagnosed at advanced or locally advanced stages, consistent with the typical clinical presentation of lung cancer in real-world settings.

**Figure 1:**
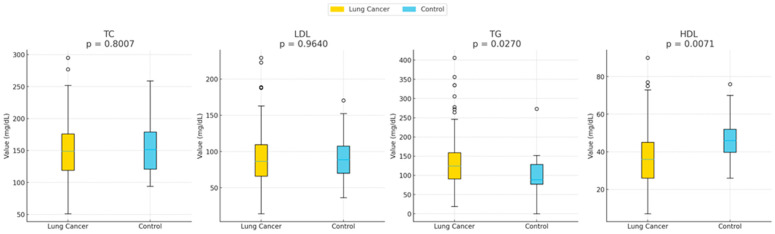
Distributions of total cholesterol (TC), high-density lipoprotein (HDL), low-density lipoprotein (LDL), and triglycerides (TG) are shown for control group (group 1) and lung cancer patients (Group 0). HDL levels were significantly lower in lung cancer individuals (*p* = 0.031). No other lipid markers showed significant differences. The boxplots represent the mean value of each lipid parameter between the two populations studied (lung cancer group vs. control group). TGs were found to be significantly higher in the lung cancer group, while HDL was found to be significantly lower.

Hypocholesterolemia was found in 61.2% of patients, while hypercholesterolemia occurred in 38.8%. Elevated LDL (>100 mg/dL) was recorded in 37.4% of included patients. Hypertriglyceridemia (TG > 150 mg/dL) was present in 36.5% and low HDL (<45 mg/dL) was observed in 77.7% of patients, indicating a pronounced alteration in lipid homeostasis. One hundred patients had significantly lower HDL levels (47.06 ± 14.4 vs. 36.01 ± 15.2, *p* = 0.006 compared with controls). All lipid parameters except TG were also lower in the cancer group. TG levels were significantly higher in NSCLC patients (137.4 ± 66.3 vs. 104.1 ± 58.7, *p* = 0.046). These results are summarized in Table 1 and illustrated in Figure 1, demonstrating clear differences in lipid profiles between groups.

### 3.2. Comparison of Lipid Levels Between Cancer Subtypes

**Figure 2:**
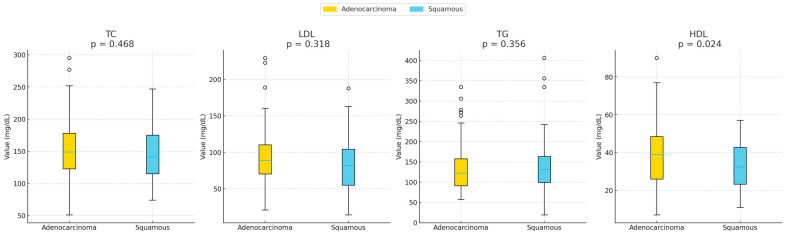
Distributions of total cholesterol (TC), high-density lipoprotein (HDL), low-density lipoprotein (LDL), and triglycerides (TG) are shown for squamous cell and adenocarcinoma lung cancer patients. HDL levels were significantly lower in individuals diagnosed with squamous lung cancer (*p* = 0.015). No other lipid markers showed significant differences. The boxplots represent the mean value of each lipid parameter between the two populations studied (squamous lung cancer group vs. adenocarcinoma lung cancer group). HDL was found statistically higher in patients with adenocarcinoma.

TG levels were higher in squamous cell carcinoma patients compared to those with adenocarcinoma (145.3 ± 83.4 vs. 133.2 ± 56.7, *p* = 0.356), while TC, LDL, and HDL were lower in the squamous group. Notably, HDL was significantly lower in squamous patients (32.43 ± 12 vs. 38.01 ± 16.5, *p* = 0.024). These findings highlight subtype-specific differences in lipid metabolism, suggesting potential biological distinctions between squamous and adenocarcinoma tumors. Data are presented in Table 2 and Figure 2.

### 3.3. Correlation Between Lipids and Age

**Figure 3:**
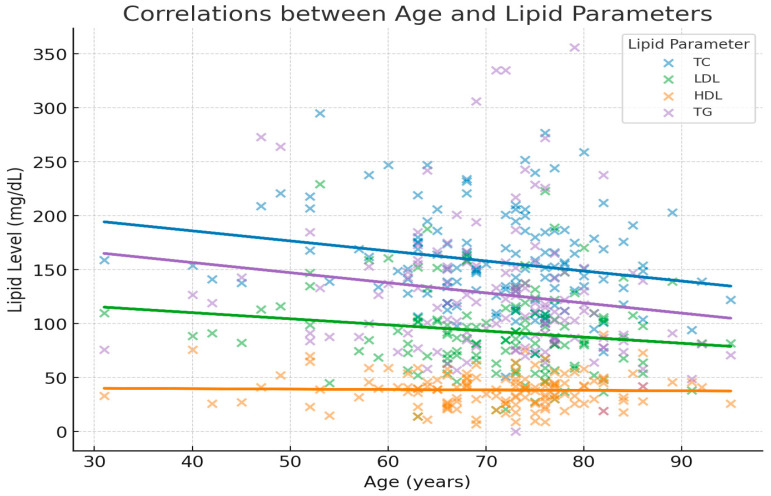
Scatterplots show the relationship between age and serum lipid levels in patients with lung cancer. Each lipid parameter is represented by a distinct color (TC = blue, LDL = green, HDL = orange, TG = purple). Regression lines indicate the general trend for each lipid across age. A weak negative correlation was observed between age and most lipid parameters, reaching statistical significance only for total cholesterol (r = −0.217, *p* = 0.013). These findings suggest that lipid levels tend to decrease slightly with increasing age, although the effect is modest.

Only total cholesterol (TC) showed no correlation with age (*p* < 0.05). The other lipid parameters showed weak, non-significant trends toward lower values with increasing age, as shown in Table 3 and Figure 3. It is noteworthy that TGs showed a weak negative correlation with a tendency towards significance.

### 3.4. Correlation Between Lipids and Biomarkers

**Figure 4:**
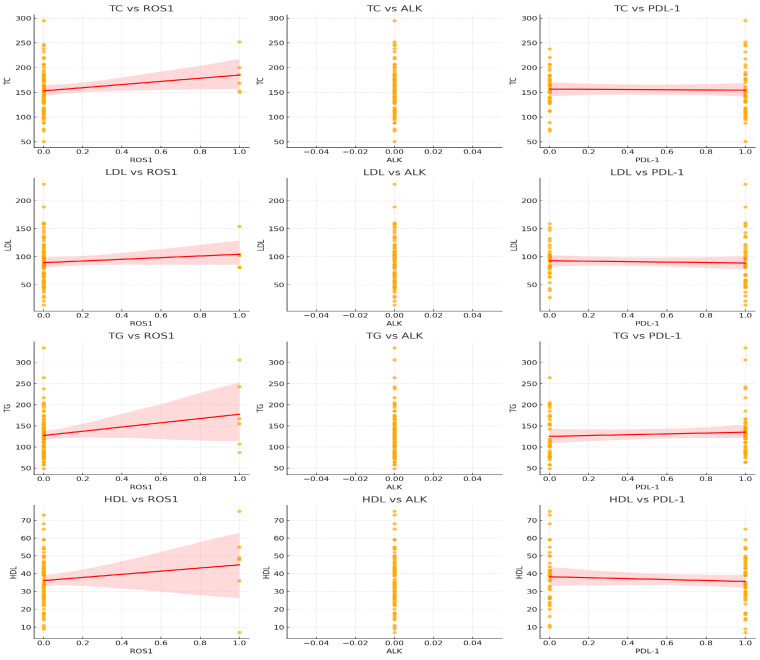
Scatterplots show statistically significant linear correlation between lipid parameters and ROS1. No other clear linear trend between lipid levels and biomarkers confirming correlation between the above-mentioned parameters was revealed. Abbreviations: TC: Total Cholesterol, HDL: High Density Lipoprotein, LDL: Low Density Lipoprotein, TG: Triglycerides, ALK: Anaplastic Lymphoma Kinase, ROS1:C-rs oncogene 1, PDL-1: Programmed Death-Ligand 1.

Correlation analysis showed a significant negative association between TG and ROS1 expression (r = −0.223, *p* = 0.004). No significant correlations were found between lipid levels and EGFR, ALK, PD-L1, or MSI. Scatterplots are shown in Figure 4.

### 3.5. Correlation Between Lipid Parameters and Outcome

The outcome was categorized as survival or death. Comparative analysis of serum lipid parameters between these two groups showed no significant differences in total cholesterol (TC), low-density lipoprotein (LDL), or triglyceride (TG) levels (all *p* > 0.05). In contrast, high-density lipoprotein (HDL) concentrations were significantly lower among patients who died compared with those who survived (mean difference = −11.1 mg/dL, 95% CI: −19.9 to −2.3, *p* = 0.014). Levene’s test indicated homogeneity of variances for all evaluated lipid parameters (*p* > 0.05). Overall, these results suggest that although most lipid parameters did not differ between outcome groups, reduced HDL cholesterol may be linked to the observed clinical or molecular differences.

## 4. Discussion

Our study demonstrates that patients with lung cancer exhibit significant alterations in lipid metabolism, most notably, reduced total cholesterol and HDL levels. These findings align with increasing evidence that cancer cells undergo metabolic reprogramming to sustain rapid proliferation, survival, and resistance to apoptosis, with lipid metabolism playing a pivotal role [4,8]. Additionally, weight loss and catabolic dominance, common features of malignancy, may further contribute to systemic lipid depletion [20]. HDL was significantly lower in lung cancer patients compared with controls, while TC, LDL, and TG were also lower but not statistically significant. Smoking, the major risk factor for this type of malignancy, is a clear contributor to these alterations. Moreover, similar results have been observed in hematologic malignancies, where cholesterol fractions were reduced [21]. These findings suggest that malignant cells utilize circulating lipids for membrane synthesis, energy production, and oncogenic signaling, leading to systemic depletion [4,20]. Low HDL may also reflect inflammation and oxidative stress, both central to lung cancer pathophysiology [9].

By subtype, SCC patients had significantly lower HDL and higher TG than ADC. Prior studies support these differences, reflecting variation in metabolic gene expression and tumor microenvironment [13,14]. Such findings imply that lipid metabolism may play distinct mechanistic roles in different histological subtypes. Hartmann et al. reported that when analyzing HDL cholesterol and BMI, they found an inverse correlation both in control subjects and in patients with lung cancer. Moreover, when they correlated total blood cholesterol with tumor diameter in lung cancer patients, they also found an inverse relationship. Altogether, this would suggest a worse prognosis in patients with high triglycerides compared to those with high blood cholesterol [22].

Beyond their systemic roles, lipids profoundly influence immune cell function within the tumor microenvironment (TME). These immune cells can exhibit either tumor-suppressive or tumor-promoting functions, and, in cancer, their lipid metabolism is frequently reprogrammed in a way that may shift the balance toward tumor immune response [23]. Altered lipid availability and metabolism can modulate T-cell exhaustion, macrophage polarization, and dendritic cell activation, thereby shaping antitumor immunity. Tumor-associated macrophages, for instance, rely on fatty acid oxidation to support an immunosuppressive phenotype, while cholesterol accumulation can impair cytotoxic T-cell responses. These mechanisms suggest that the systemic dyslipidemia observed in our lung cancer cohort may reflect, or even drive, immune dysregulation within the TME. Integrating lipidomic profiling with immune biomarkers in future studies could clarify this relationship and reveal new metabolic targets to enhance immunotherapy efficacy [24].

**Figure 5:**
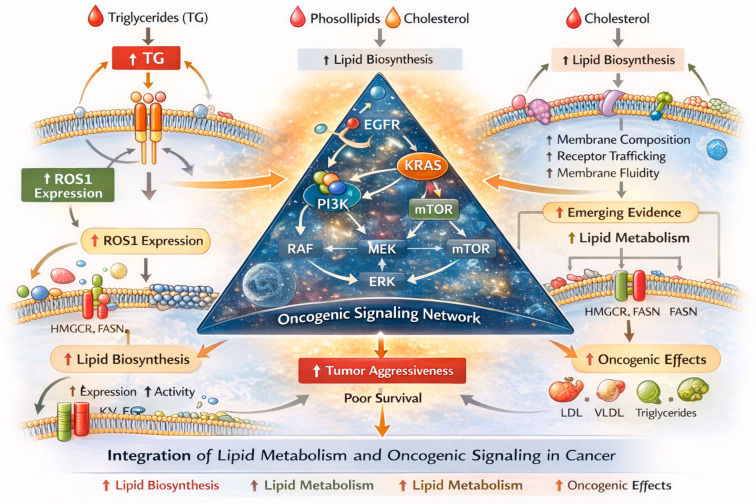
Alterations in Lipid Metabolism and Oncogenic Signaling in Cancer.

In addition to non-small cell lung cancer, metabolic alterations—including those involving lipid pathways—have been described in small cell lung cancer (SCLC). Recent proteomic profiling studies have shown that lipid metabolism, particularly phospholipid metabolism, plays a significant role in SCLC tumor behavior, including recurrence and metastasis, suggesting that aberrant lipid handling may contribute to aggressive biology in this subtype. Furthermore, serum lipid parameters such as HDL and LDL cholesterol have been identified as independent prognostic factors for disease-free survival in patients with SCLC, indicating clinical relevance of lipid alterations in this context. Metabolomic analyses in SCLC patients have also revealed significant disturbances in lipid-related metabolic pathways, supporting the concept that lipid metabolism is dysregulated in this histological subtype [25]. Changes in lipid profile parameters appear not to be limited to patients with lung cancer. Similar alterations have also been reported in patients with renal carcinoma. Cancer cells employ several adaptive strategies to sustain elevated intracellular cholesterol levels. These include enhanced endogenous cholesterol synthesis, regulated by sterol response element-binding proteins (SREBPs), as well as increased uptake of LDL cholesterol. Elevated sterol levels activate sterol-sensing liver X receptors (LXRs), which, in turn, promote cholesterol efflux by inducing the expression of cholesterol transport proteins [26]. Additionally, LXRs downregulate LDL cholesterol uptake by stimulating the transcription of an E3 ubiquitin ligase known as the inducible degrader of LDLR [27]. Collectively, these mechanisms highlight the SREBP and LXR pathways as promising therapeutic targets in cancer treatment (Figure 5).

**Figure 6:**
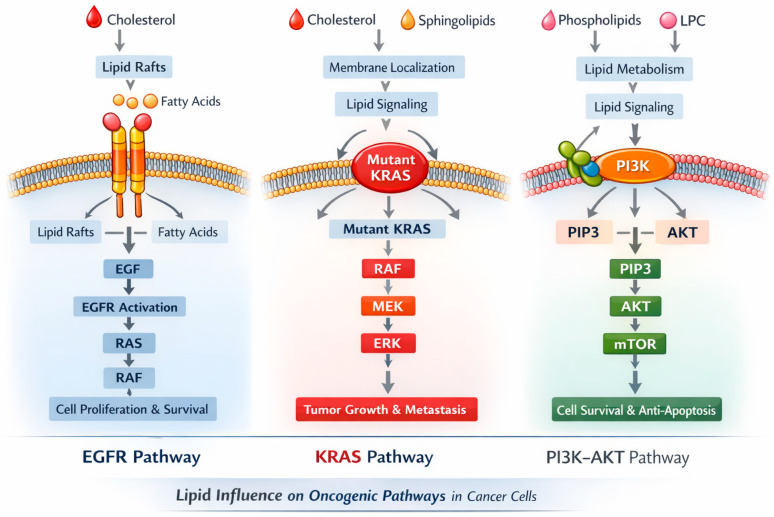
Lipid influence in Oncogenic Pathways in Cancer patients.

Our analysis also showed a negative association between TG and ROS1 expression, suggesting interactions between lipid metabolism and oncogenic drivers. Cancer cells exhibit markedly elevated rates of lipid synthesis—especially of phospholipids (PLs) and cholesterol—through the upregulation of both the expression and activity of key enzymes involved in their biosynthetic pathways [28]. This supports emerging evidence linking lipid pathways with tumor aggressiveness [15,29]. Elevated TG and reduced HDL have also been identified as prognostic markers for worse survival in NSCLC [30]. Beyond their metabolic role, lipids may influence oncogenic signaling by modulating membrane fluidity, lipid raft composition, and receptor trafficking, thereby affecting pathways such as EGFR, KRAS, and PI3K–AKT. This integration of metabolic and signaling networks underscores the complexity of cancer progression and highlights the need for multitopic approaches to fully understand these interactions (Figure 6).

The clinical implications are important. Lipid parameters, particularly HDL, may serve as accessible biomarkers of metabolic alterations in lung cancer. Associations with molecular biomarkers raise the possibility that lipid profiling could complement genomic testing in guiding therapy. For instance, baseline lipid profiles might help identify tumors with specific metabolic dependencies, which could be exploited therapeutically. Furthermore, interventions that modulate lipid metabolism—such as statins, LXR agonists, or inhibitors of SREBP activation—could potentially synergize with targeted or immunotherapies. Several preclinical studies have suggested that lowering intracellular cholesterol can impair cancer cell proliferation and sensitize tumors to cytotoxic agents, while others indicate that manipulating lipid efflux pathways can influence the tumor immune microenvironment by altering macrophage and T-cell function [30,31].

The observed association between lower HDL levels and increased mortality likely reflects a complex interplay between tumor-specific biological processes and systemic host factors. Beyond potential direct effects of lipid metabolism on tumor growth and progression, reduced HDL levels in patients with lung cancer may also be influenced by cancer-related inflammation, metabolic derangements, cachexia, and overall frailty, particularly in advanced stages of disease. Kim et al. showed a U-shaped association between baseline TC levels and risk of mortality in a large-scale longitudinal analysis of 59,217 patients with cancer, with low and high levels associated with an elevated risk. Low LDL-C levels (first and second centile groups) were associated with increased risk of death, but no similar risk increase was observed among patients with higher LDL-C (sixth and seventh centile groups). As expected, a decrease in HDL-C values was associated with an increased risk of death in patients with cancer [32]. In this context, HDL may function not only as a marker of tumor biology but also as an indicator of the systemic response to malignancy. Therefore, the prognostic significance of HDL in our cohort should be interpreted cautiously, as causality cannot be inferred.

Furthermore, the low-density lipoprotein receptor (LDLR), a single-pass transmembrane receptor, is essential for mediating cellular cholesterol uptake and maintaining cholesterol homeostasis. Recent studies have highlighted LDLR as a key metabolic target involved in limiting the progression of various cancers, such as pancreatic ductal adenocarcinoma, breast cancer, liver cancer, and bladder cancer. In addition, EGFR signaling has been shown to modulate LDLR expression through an SREBP-1–dependent mechanism [28]. This further strengthens the link between oncogenic signaling and lipid metabolism, suggesting that tumors may exploit growth factor pathways to increase lipid availability for anabolic processes [31].

This study has several strengths, including the inclusion of a well-defined, real-world cohort with comprehensive molecular profiling and standardized lipid measurements. To our knowledge, this is one of the few studies to examine associations between lipid parameters and specific oncogenic drivers in NSCLC. Our study has limitations. Many patients included in the analysis were not hospitalized at our center but instead were referred to by other hospitals solely for bronchoscopy or molecular testing. We also recognize that several potential confounders (including lipid-lowering medications, metabolic comorbidities, diet, and lifestyle factors) were not consistently available and therefore could not be incorporated into adjusted models. As a result, several clinically relevant variables—such as BMI, performance status, comorbidity burden, disease stage, statin treatment and socioeconomic characteristics—were not consistently available and could not be reliably incorporated. Its retrospective, single-center design may introduce bias, and the limited sample size (*n* = 160), particularly in certain driver mutation subgroups, undermines the statistical power. Lipid measurements were not standardized for fasting status, potentially affecting values. Longitudinal follow-up was also lacking. Furthermore, in our study, molecular data were limited, so detailed analyses linking lipid levels to specific genetic alterations within each subtype were not feasible. Despite these limitations, the findings provide real-world evidence of the interplay between lipid metabolism, histology, and molecular biomarkers.

In a real-world, pre-treatment cohort of patients with NSCLC and comprehensive molecular profiling, routine serum lipid parameters revealed both histology-specific patterns (lower HDL levels in squamous cell carcinoma compared to adenocarcinoma) and a novel inverse association between triglycerides and ROS1 positivity. Unlike previous studies that have focused on lipidomic analyses or therapy-induced lipid profile alterations, this study identifies a baseline, low-cost lipid signal that may reflect a metabolic phenotype of ROS1-rearranged tumors. Even if we observed an inverse association between triglyceride levels and ROS1 expression, the number of ROS1-positive cases in our cohort was relatively small. This result should therefore be considered exploratory and hypothesis-generating rather than confirmatory. Larger, well-characterized cohorts are needed to validate this observation and to investigate whether a true biological relationship exists between triglyceride metabolism and ROS1-driven lung cancer. In contrast, no pre-treatment lipid differences were observed between EGFR- or ALK-positive and negative patients, suggesting that common lipid markers are likely more useful in prognostic or treatment-response settings rather than for diagnostic detection of molecular alterations.

In conclusion, lung cancer is associated with distinct lipid alterations, primarily reduced HDL, with significant subtype differences. Correlations with oncogenic drivers highlight the potential interplay between lipid metabolism and tumor signaling. These observations support the idea that metabolic reprogramming is not merely a bystander effect but an integral part of tumor biology. Prospective studies are warranted to validate these findings, clarify their prognostic role, and explore the therapeutic potential of targeting lipid metabolism in lung cancer.

## 5. Conclusions

In conclusion, these findings support the concept that common, low-cost lipid parameters—particularly high-density lipoprotein—may reflect clinically meaningful metabolic and inflammatory changes in lung cancer and could be explored further as adjunct biomarkers in research settings. However, interpretation of our data should remain cautious because of the retrospective design, incomplete information on key confounders (such as body mass index, comorbidities, stage, inflammation markers, and fasting status), and limited numbers within molecular subgroups.

Larger prospective studies with standardized sampling and comprehensive clinical annotation are needed to validate the observed associations, clarify whether lipid alterations are merely markers of disease burden or contribute to tumor biology, and determine whether integrating lipid profiling with molecular testing can improve risk stratification or inform future therapeutic strategies targeting lipid metabolism.

## Figures and Tables

**Table 1:** Comparison of all lipid measurements between lung cancer patients and healthy controls.

Lipid Parameter (mg/dL)	Lung Cancer Patients (n = 160) Mean ± SD	Healthy Controls (n = 100) Mean ± SD	*p*-Value
**Total Cholesterol (TC)**	152.5 ± 44.8	156.9 ± 47.9	0.800
**LDL Cholesterol (LDL)**	91.5 ± 39.0	92.3 ± 38.2	0.960
**Triglycerides (TG)**	137.4 ± 66.3	104.1 ± 58.7	0.027
**HDL Cholesterol (HDL)**	36.0 ± 15.2	47.1 ± 13.4	0.007

Abbreviations: total cholesterol (TC), low-density lipoprotein (LDL), high-density lipoprotein (HDL), and triglycerides (TG). Control: 0 refers to patients with lung cancer and 1 refers to healthy individuals.

**Table 2:** Comparison of all lipid measurements between adenocarcinoma and squamous cell lung cancer in patients with NSCLC.

Lipid Parameter (mg/dL)	Adenocarcinoma Mean ± SD	Squamous Cell Carcinoma Mean ± SD	*p*-Value
**Total Cholesterol (TC)**	153.8 ± 47.8	148.4 ± 39.5	0.468
**LDL Cholesterol (LDL)**	93.3 ± 40.5	86.2 ± 36.3	0.318
**Triglycerides (TG)**	133.2 ± 56.7	145.3 ± 83.4	0.356
**HDL Cholesterol (HDL)**	38.0 ± 16.5	32.4 ± 12.0	0.024

**Table 3:** Correlation between lipid parameters and age.

Parameter	r	*p*-Value	Interpretation
**Total Cholesterol (TC)**	−0.217	0.013	Weak negative correlation, statistically significant
**LDL**	−0.151	0.084	Weak negative, not significant
**HDL**	−0.027	0.761	No correlation
**Triglycerides (TG)**	−0.159	0.068	Weak negative, trend toward significance

## Data Availability

The data that support the findings of this study are available from the corresponding author upon reasonable request.
